# Overview of emerging semiconductor device model methodologies: From device physics to machine learning engines

**DOI:** 10.1016/j.fmre.2024.01.010

**Published:** 2024-02-06

**Authors:** Xufan Li, Zhenhua Wu, Gerhard Rzepa, Markus Karner, Haoqing Xu, Zhicheng Wu, Wei Wang, Guanhua Yang, Qing Luo, Lingfei Wang, Ling Li

**Affiliations:** a State Key Lab of Fabrication Technologies for Integrated Circuits, Institute of Microelectronics, Chinese Academy of Sciences, Beijing 100029, China; b University of Chinese Academy of Sciences, Beijing 100049, China; c Global TCAD Solutions, Vienna 1010, Austria; d Peng Cheng Laboratory, Shenzhen 518066, China

**Keywords:** Compact model, Machine learning, Artificial Neural Network, Technology Computer-Aided Design, Design technology co-optimization

## Abstract

Advancements in the semiconductor industry introduce novel channel materials, device structures, and integration methods, leading to intricate physics challenges when characterizing devices at circuit level. Nevertheless, accurate models for emerging devices are crucial for physics-driven TCAD-to-SPICE flows to enable the increasingly vital design technology co-optimization (DTCO). Particularly for ultra-scaled devices where quantum effects become significant, this led to the introduction of empirical model parameters and a disconnection to manufacturing processes. To catch up with these developments, an alternative to the traditional *white-box* modeling methods has attracted much attention: machine learning-assisted compact modeling (MLCM). These *black-box* methods target towards general-purpose modeling of complex mathematics and physics through training of neural networks on experimental and simulated data, generating an accurate closed-form mapping between output characteristics and input parameters for fabrication process and device operation. To address this new trend, this work provides a comprehensive overview of emerging device model methodologies, spanning from device physics to machine learning engines. By analyzing, structuring, and extending distributed efforts on this topic, it is shown how MLCM can overcome limitations of traditional compact modeling and contribute to effective DTCO to further advance semiconductor technologies.

## 1 Introduction

**Fig. 1:**
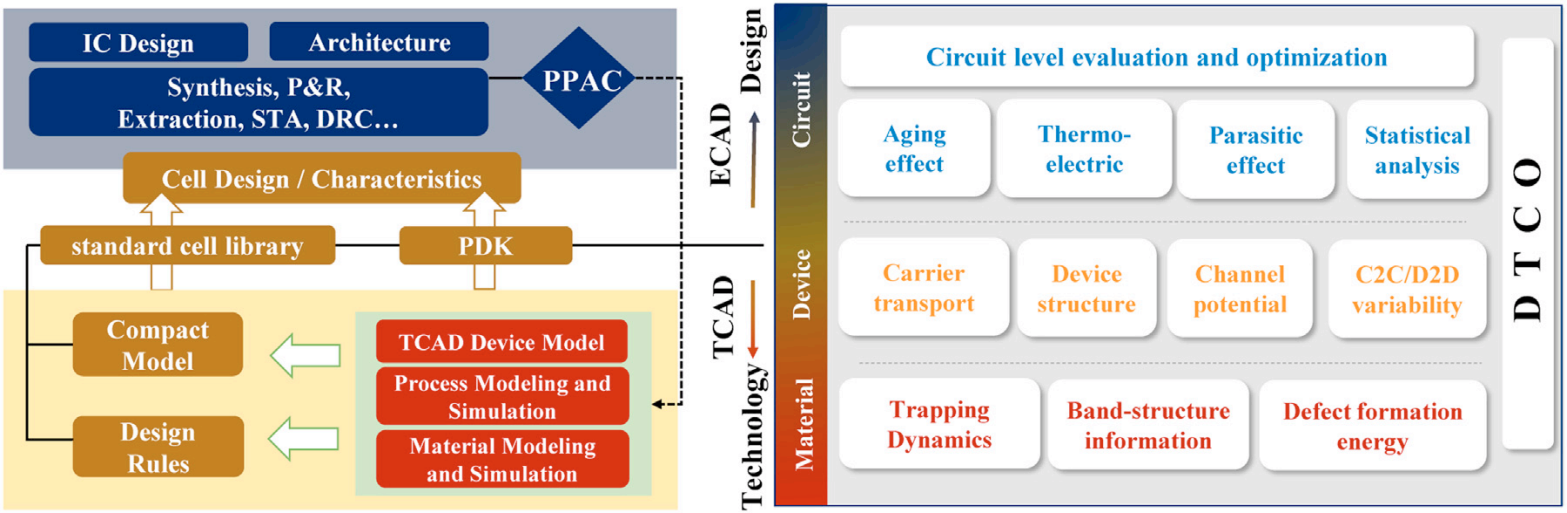
**Schematic DTCO flow for evaluation of the power, performance, area, and cost (PPAC) metrics: semiconductor device models, which correctly capture the physics of the carrier transport, variability, reliability, etc., serves as the cornerstone of bridging electronic computer-aided design (ECAD) and technology computer-aided design (TCAD)**.

In design technology co-optimization (DTCO) flows, semiconductor device models are the bridge between the device fabrication and the circuit design, as shown in Fig. 1. Enabling circuit simulations with accurate device models is important for correct analysis of trade-offs of efficiency and accuracy, facilitating optimization of PPAC of circuits.

With TCAD-to-SPICE techniques, technology-dependent parameters of device models are extracted based on TCAD results, and then are implemented into the circuit simulator. To conduct realistic assessment of the represented IC technology in ECAD, many important reliability issues, such as parasitic effects, quantum mechanisms, bias temperature instability (BTI), hot carrier degradation (HCD), gate leakage, and kink effects, have to be incorporated in TCAD simulations. Therefore, an excellent TCAD-to-SPICE model serves as the link between the state-of-the-art technology and integrated circuit design, encompassing device geometry, bias voltage, temperature, DC, AC, radio frequency (RF), noise and so on.

***Traditional physics-driven TCAD*** plays a critical role in state-of-the-art semiconductor device design and pathfinding. However, as semiconductor devices scale down to the 5 nm technology node and beyond, quantum physics induced effects are inevitable. This leads to considerable additional complexity in TCAD theory and methodology, also causing enormous computational costs. To improve the predictability and the computational efficiency of TCAD simulations, there have been many studies on developing advanced physical models beyond drift-diffusion (DD) frameworks [1], as well as incorporating high performance computing [2].

***Traditional physics-driven compact models*** are mainstream for circuit CAD and have continuously evolved since the first compact model (Ebers-Moll, EM) for bipolar transistors in 1954 [3]. This physics-based model has limited accuracy due to various approximations and the more accurate Gummel-Poon compact model was proposed based on integrated charge control relations in 1970 [4]. Subsequently, the rapid development of Simulation Program with Integrated Circuit Emphasis (SPICE) and MOS technology promoted a transition from simple basic compact metal-oxide-semiconductor (MOS) field-effect transistor (FET) models to today's sophisticated models over the past 50 years.

**Fig. 2:**
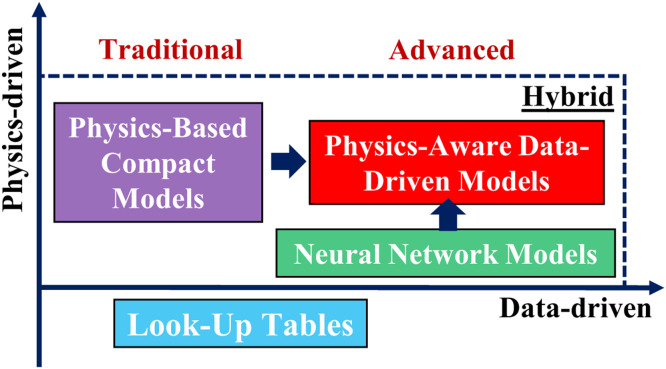
**Schematic of usage of physics and data for different modeling methods**.

***Artificial intelligence* (*AI***) ***technology*** has been developed recently to accelerate the development of TCAD-to-SPICE methodology. Unlike the physics-driven modeling, as shown in Fig. 2, data-driven modeling, including look-up tables (LUT) and machine learning-assisted compact modeling (MLCM), is a *black-box* method with detailed physical parameters transparent to IC designers and model developers. This method refrains from computational complexities, but lacks physics, affecting convergence of very large-scale circuit (VLSI) simulation. For rapid implementation of measured device data into circuit simulations, the table-based model, as known as LUT, is generally used. Besides characterizing I-V, a generic approach for capturing process variations in LUT-based models was proposed for circuit-level analysis [5]. The model is data-sensitive, and its accuracy is closely related to the noise, data-size, and interpolation methods. Since the LUT is originated from experimentally measured data-set and is not physically explainable, the lack of predictive capability indicates the limited applicability to the path-finding study when implementing such approach to advanced technology and the process development is still in early stage. Another data-driven compact modeling approach is the Artificial Neural Networks (ANN)-based model via replacing tables and interpolation schemes. Compared to LUT, ANN-based model may exhibit better scalability, generalization, smoothness, etc. [6]

## 2 Device physics engine model development

### 2.1 Brief of traditional TCAD models

**Fig. 3:**
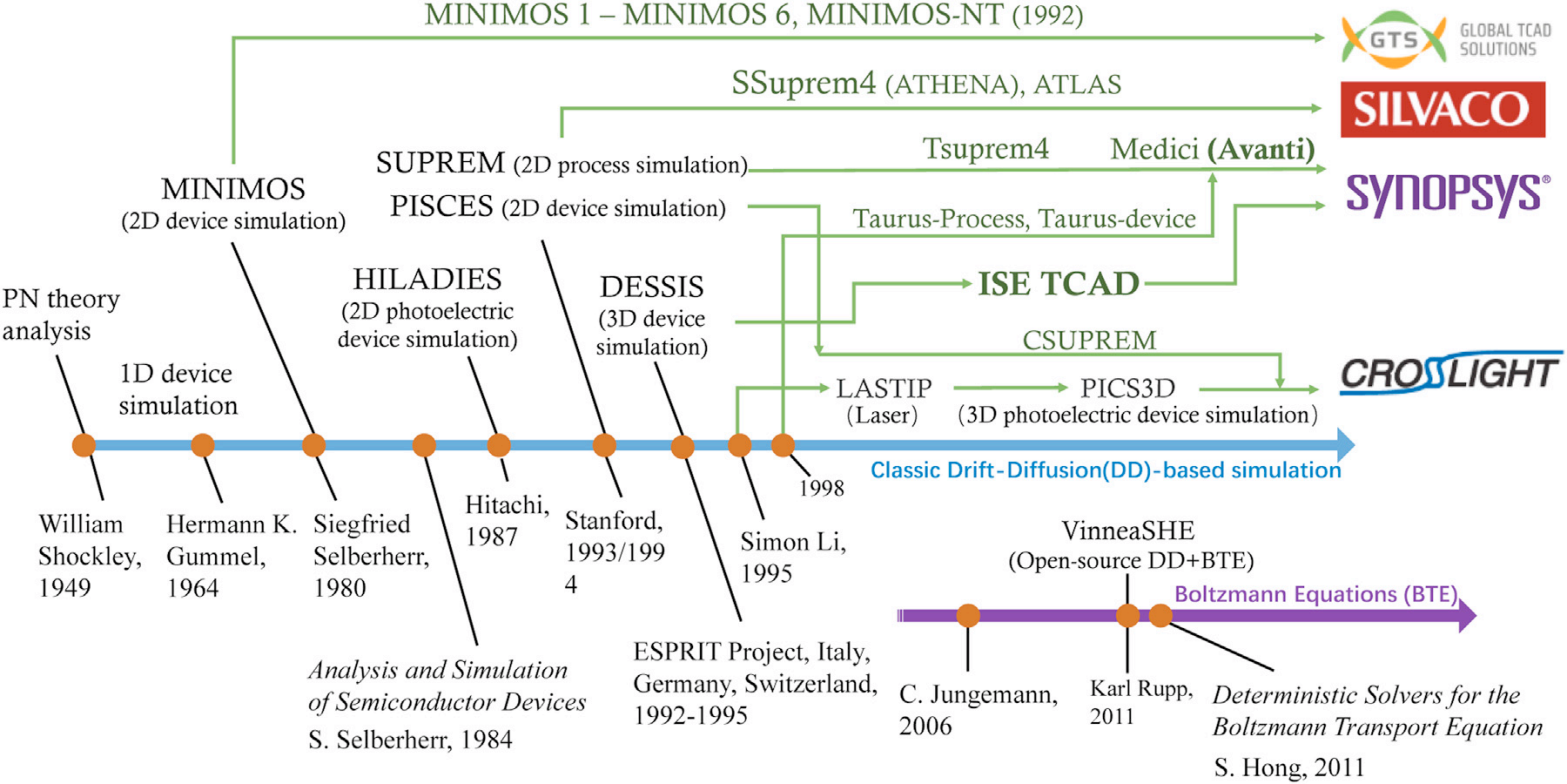
**The evolution of TCAD based on drift-diffusion and Boltzmann equation**.

Since the PN theory analysis was proposed in 1949, TCAD has evolved for more than seventy years now (Fig. 3). In the 1960s, Bell Labs and IBM completed simulations for the diode device in a specific device structure configuration. In 1964, Gummel reported a self-consistent iterative method for one-dimensional transistor simulation calculation [7]. Based on this the Gummel-Poon model proposed by Gummel and Poon in 1970, provided the fundamentals for triode model development [4]. With the development of field-effect-controlled devices, P. Cottrell and E. Buturla proposed a two-dimensional finite element cross analysis method [8] in 1975, benefiting the algorithm development for multi-dimensional device simulation tools. As the one-dimensional simulations did not meet the requirement to solve complicated coupling issues due to device geometry scaling encountered later in the 1970s, Siegfried Selberherr developed the MINIMOS tool to realize the simulation analysis of two-dimensional field effect transistors [9]. This was followed by three-dimensional device simulation tools (e.g., MINIMOS-5 and MINIMOS-6) in the 1980s. This development played an important role in understanding and suppressing the short-channel effect and optimizing the doping profiles due to device miniaturization. Moreover, from the late 1970s to the mid-1980s, D. A. Antoniadis developed the SUPREM series of tools to realize integrated circuit process simulation [10] and M. R. Pinto developed the multi-dimensional device simulator PISCES IIB [11].

**Fig. 4:**
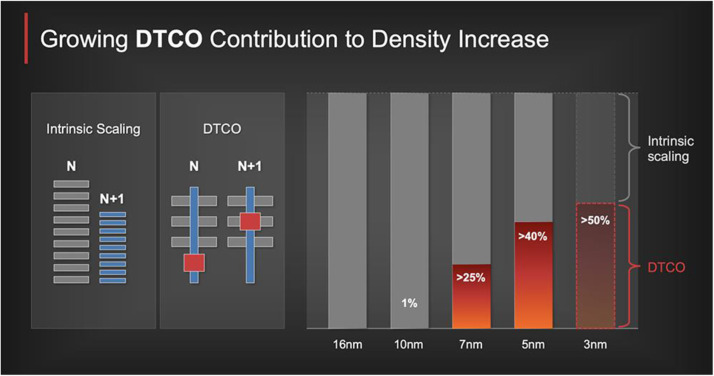
**Trend of cumulative relative contribution to gate density increase by DTCO and traditional “intrinsic” scaling show growing DTCO contribution to density increase**[12].

In the past 20 years, in view of the complexity and high cost of industrial production processes, the exploration of device structure design and performance optimization assisted by TCAD has become more important. Thanks to early TCAD development, several mainstream TCAD tools, including Global TCAD Solutions, Crosslight TCAD, Silvaco TCAD, Sentaurus TCAD etc., emerged and started raising the contribution of DTCO to density increase (Fig. 4). As of 2023, with aggressive scaling, the number of transistors can reach hundreds of billions per chip, and high-speed device simulation algorithm should be further explored. However, when device-scaling approaches atom-level, traditional TCAD encounters the bottleneck of incorporation of quantum mechanisms, possibly leading to a paradigm shift from traditional device physics to machine learning engines.

### 2.2 Brief of traditional compact models

**Fig. 5:**
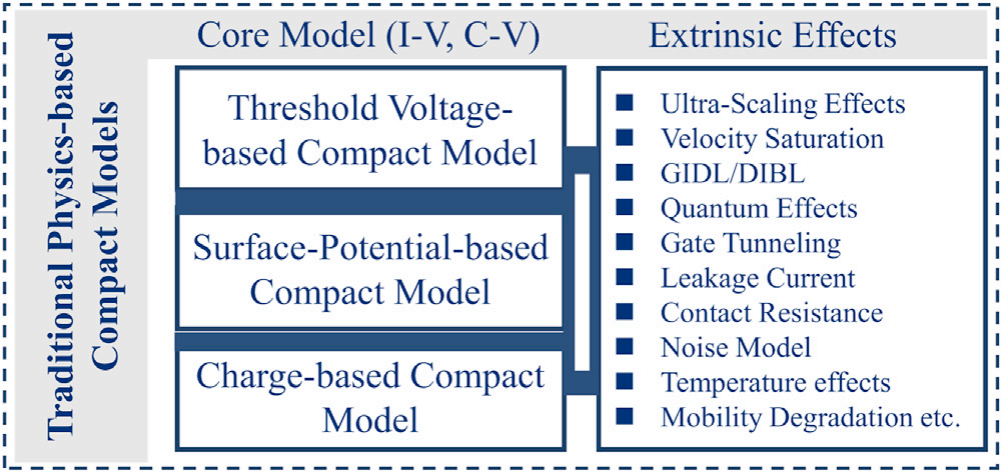
**Schematic of core model and extrinsic effects**.

**Fig. 6:**
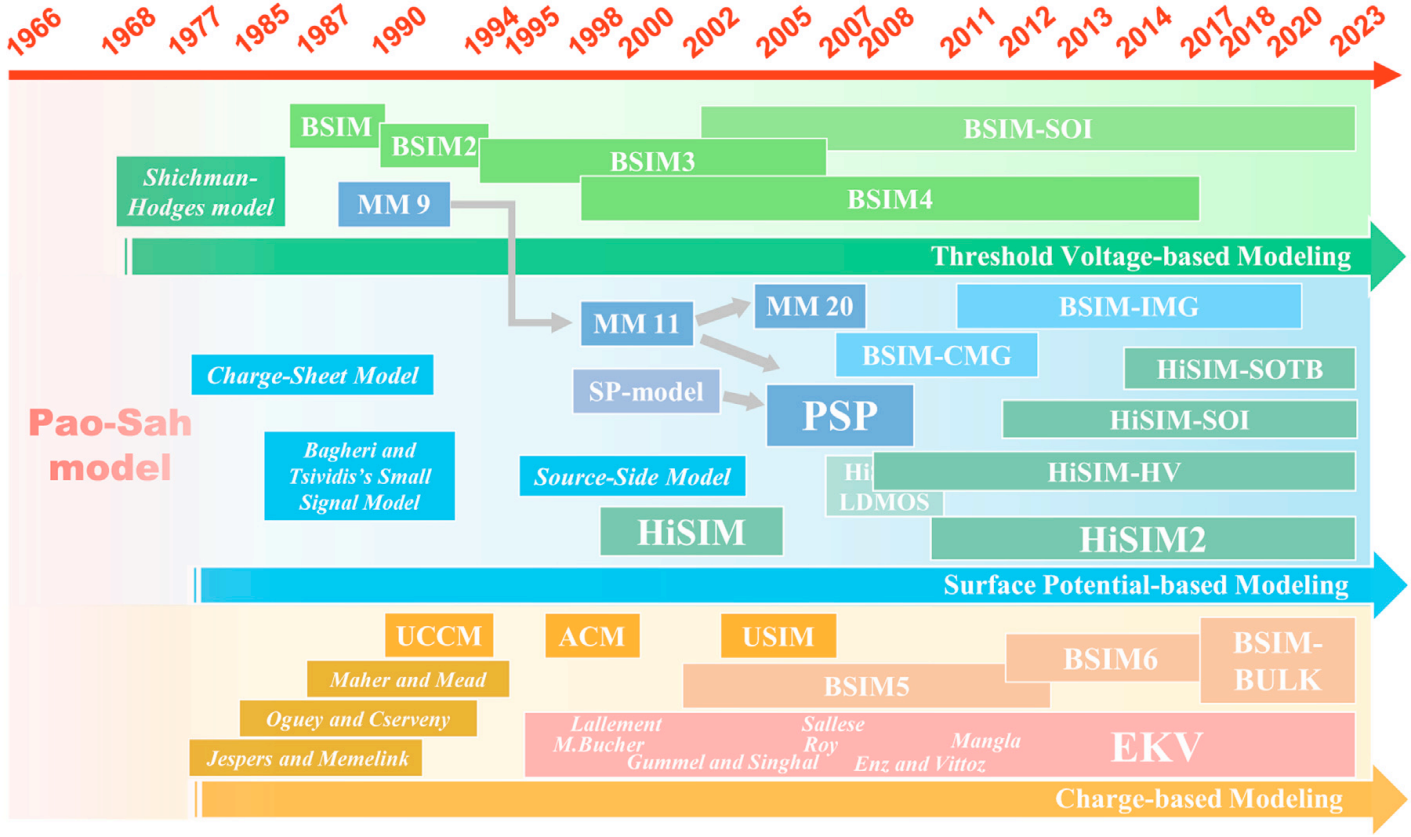
**Evolution chart of the mainstream compact models:*****ϕ***_**s**_**-based,*****V***_**th**_**-based, and*****Q*****-based.**

The Pao-Sah model, introduced in 1966 [13], defines the drain-source current *I*_ds_ as

(1) $$\begin{array}{c} I_{\mathrm{ds}}\;=\;-\mu WQ_{i}\cdot \frac{dV_{c}}{dy} \end{array}$$

where *W* represents the channel width, *μ* the carrier mobility, *Q*_i_ the inversion charge density, and *V*_c_ the channel potential. The Pao-Sah model describes the drift and diffusion behavior and is valid in all operation regions. It points out an important physical parameter called the surface potential (*ϕ*_s_), and the threshold voltage (*V*_th_) can be defined as a particular value of surface potential characterizing the operation mode. Such a model is quite complicated, and *ϕ*_s_ can be only solved numerically requiring iteration algorithm from the implicit relation. As a result, efforts to search for accurate and computationally efficient compact models for ECAD have been devoted since the late 1960s via different approaches. Such models can be described as threshold voltage-based (*V*_th_-based), surface potential-based (*ϕ*_s_-based), and charge-based (*Q*-based) (Figs. 5 and 6).

#### 2.2.1 Threshold voltage-based modeling

The current can be calculated analytically by using *V*_th_ as the boundary between different operation modes (weak inversion region and strong inversion region), which is so-called *V*_th_*-*based compact model. The advantage is to circumvent the iterative computation of *ϕ*_s_ and relate the input voltage directly to current equation, contributing to the widely used parabolic form in 1968 [14]. It is expressed in

(2) $$\begin{array}{ccc} I_{\text{ds}}\; & = & \;K_{1}\cdot {\left(V_{\text{gs}}-V_{\mathrm{th}}\right)}^{2}\cdot \left(1+l\cdot \left|V_{\text{gs}}-V_{\mathrm{gd}}\right|\right);\\ K_{1}\; & = & \;\mu \cdot \frac{C}{2}\cdot \frac{W}{L}\;\left(\text{above}\;\text{knee}\right) \end{array}$$

In 1974, MOSFET scaling rule was proposed [15]. The continuous scaling trend results in complicated device physics and inaccuracy of *V*_th_-based compact model. Thus, for better understanding short channel effects, the Berkeley Short-channel IGFET Model, commonly known as BSIM, was developed in 1987 [16] and then followed by improved models, such as the BSIM2 in 1990 and the BSIM3 in 1994. Besides, Philips Laboratories also proposed the MOS Model 9 (MM9) compatible to mainstream CAD tools, featuring well-behaved hyperbolic expressions and less model parameters.

#### 2.2.2 Surface potential-based modeling

Referring to Pao-Sah equation, it is basically a surface potential based current model. Hence, to retain more physics information, the *ϕ*_s_-based compact approach is preferred but facing a big challenge to derive an analytical surface potential at the end of channel (i.e., Drain-terminal *ϕ*_sd_ or Source-terminal *ϕ*_ss_). Based on the early MOSFET charge-sheet model proposed by J.R. Brews in 1977 [17], a simple *ϕ*_s_-based form [18] is given by

(3) $$\begin{array}{c} I_{\text{ds}}\;=\;\mu \cdot C_{\text{ox}}\cdot \frac{W}{L}\left[\left(V_{\text{gs}}-V_{\text{fb}}+2V_{\text{t}}\right)\cdot \left(\phi_{\text{sd}}-\phi_{\text{ss}}\right)-\frac{1}{2}\left(\phi_{\text{sd}}^{2}-\phi_{\text{ss}}^{2}\right)\right] \end{array}$$

The advantage of such a model is close to mechanisms behind the devices. That is, the terminal charges, currents, and derivatives are functions of the *ϕ*_s_. By incorporating accurate *ϕ*_s_, researchers work on solving *ϕ*_s_ from the implicit equations directly and obtain considerable progress during 1980s. In 1985, Bagheri and Tsividis proposed the efficient algorithm by Schroder series method, which is extensively used for modern thin-film transistors [18,19]. In 1995, source-side-only model is proposed featuring excellent demonstration of transition between different working modes for silicon-on-insulator (SOI) devices. This approach offers a compromise between accuracy and efficiency, by avoiding solving the drain-side *ϕ*_s_. Unlike source-side-only model, Hiroshima-University STARC IGFET Model (HiSIM) [20] is proposed by solving two-side *ϕ*_s_ with both high accuracy and efficiency. This model serves for scaled devices including several important physical effects by modifying the potential equations inducing *V*_th_ shift with less parameters, and is followed by the SP-model proposed by The Pennsylvania State University. Besides, Philips semiconductors also propose MOS Model 11 (MM11) for scaled devices based on a linearization of the inversion charge as a function of *ϕ*_s_. By merging features of MM11 and SP, PSP model was jointly developed by The Pennsylvania State University and Philips in 2005. For extreme scaling behaviors in multi-gate devices, surface potential method has also been applied into BSIM models, such as BSIM-IMG (Independent Multi-Gate) [21], BSIM-CMG (Common Multi-Gate) [22].

#### 2.2.3 Charge-based modeling

The *Q-*based models are developed in terms of the inversion charge density (*Q*_i_) at two ends of channel. The challenge, similar to *ϕ*_s_, is to calculate the charge density efficiently based on an implicit equation. This alternative approach is proposed due to increasing complexities of *V*_th_*-*based models and difficulty in analytical solution of *ϕ*_s_*-*based models. In 1990, a unified charge control model (UCCM) [23] is proposed and provides fundamentals relating charge densities to terminal voltages. Based on UCCM, a current-voltage form [24] is given by

(4) $$\begin{array}{c} I_{\text{ds}}\;=\;q\cdot \mu \left(\alpha \right)\cdot \frac{W}{L}\left[\eta {\text{V}}_{\text{t}}\cdot \left(n_{s}-n_{d}\right)-\frac{a}{2}\left(n_{s}^{2}-n_{d}^{2}\right)\right] \end{array}$$

where *n*_s_ and *n*_d_ are the electron concentration in source and drain ends of the channel.

Subsequently, several charge-based compact models, such as unified charge control model (USIM) [25], “Advanced Compact MOSFET” model (ACM) [26], EKV (Enz, Krummenacher, Vittoz) MOSFET model [27], BSIM5, BSIM6 etc., have been reported. However, for extremely scaled multi-gate devices, the *ϕ*_s_*-*based models are superior in device physics description compared to other approaches.

## 3 Bottlenecks of physics-engine TCAD-to-SPICE in post-Moore era

The physics-engine compact modeling is a typical Poisson's equation-centric *white-box* method. Most core device characteristics are related to potential profile in MOSFETs and obtaining an analytical relation between MOSFET characteristics and terminal voltages under simplifying assumptions is fundamental to those compact models. Moreover, such models should meet basic requirements including symmetry, continuity, scalability, and efficiency with as few parameters as possible. However, when it comes to *post-Moore era*, several challenges emerge as bottlenecks of further improvement of physics-driven compact modeling methodology stratifying above requirements.

***Near-threshold Modeling:*** For today's near-threshold circuit design, transition region models become increasingly important. However, the physical approach faces a challenge of modeling a transition between sub-threshold to above-threshold region. To optimize the power dissipation and guarantee the performance, a typical near-threshold drain current model was proposed [28], without the delay and power parameters. In 2010s, near-threshold models with a fitting method, inverse charge model or essential design parameters are reported for calculation of delay and energy [29,30].

***Atomic physics and quantum correction:*** As the geometric scaling of Si-based transistors approaches its physical limit, traditional device physics need to be extended by atomic physics. For example, quantum confinement effects emerge in the extremely scaled nanosheet-based gate-all-around FET (GAA-FET) [31,32]. Thus, the conventional Poisson's equation is coupled with Schrödinger equation (S-P). The density state function at the surface of an ultra-thin body of silicon-based channel will depend on the channel thickness, as established for SOI-FETs. For advanced nanosheet GAA-FETs, it is more complicated and depends on both thickness and width of the silicon nanosheets. When considering the geometric variability of multi-nanosheets, the threshold voltage variation will be a critical yet challenging aspect for compact modeling. That makes quantum corrections of traditional model essential for those applications. However, complicated coupled parameters in S-P equations make it difficult to obtain analytical expressions using the traditional compact modeling methodology. To tackle this issue, alternative methods such as Bohm's potential and sub-bands have been established to predict profiles of the potential and carrier density due to quantum effects [31].

***Increasing complexity:*** Complicated physics contribute to increasingly non-physical parameters for fitting purpose. A plenty of fitting parameters are introduced in empirical formulations, leading to increasing complexity and challenging parameter extraction. Such non-physical parameters enlarge the gap between process and design.

***Extreme environment:*** MOSFETs operate in extreme environment for specific applications, such as aerospace detecting, quantum computing, and cryogenic high-performance-computing (HPC) down to temperatures of about 4.2 K. Effects of structural disorders will be more pronounced in low temperature regions. However, most compact models today do not extend correctly to cryogenic condition and mobilities, threshold voltages, and noise have to be reconsidered [33]. Thus, compact models in extreme environment still require substantial effort.

***Reliability Characterization:*** For advanced devices, reliability issues including ultralong-term aging prediction, statistical variability analysis, and performance degradation particularly in the context of cycle-to-cycle and device-to-device reliability are increasingly challenging from device modeling point of view, and even more so for compact modeling.

***Hetero-integration IC:*** The Heterogeneous Integration Roadmap (2021 Edition) points out four disciplines of compact modeling which should be compatible with co-design tools. In terms of monolithic 3D integration, specific challenges of compact modeling for introducing back-end-of-line process compatible novel oxide semiconductor devices, such as ferroelectric FETs (FeFETs) [34,35], resistive RAM [36] and In-Ga-Zn-O (IGZO) FETs [19,37], arise due to different material properties, working principles, and reliability mechanisms. Material beyond silicon is an issue in recent TCAD simulations due to complicated disorder induced transport behaviors or band-structures and device compact models are still far from standardization.

## 4 Machine learning engine TCAD development

As an alternative to aforementioned physics-based models, machine learning is introduced into TCAD and implemented not only in atomistic calculations but also device- and circuit-level simulations.

### 4.1 Atomistic calculations with ML

Density functional theory (DFT) and DFT-based ab-initio TCAD are powerful tools to calculate and analyze the properties of materials in nanoscale semiconductor devices, especially when the critical dimension of devices reaches atomistic scale and quantum effects are not negligible. However, DFT is quite computationally expensive with a trade-off between accuracy and efficiency. Therefore, novel machine learning approaches are introduced where neural network (NN) show promising results for capturing nonlinearities and predictive value. One example is to solve the Schrodinger equation and achieve almost exact solutions for molecules with up to 30 electrons with an efficiency outperforming state-of-art variational ansatz with high accuracy [38]. In another example, NN performs as a wave-function ansatz for the many-electron system in ab-initio calculation and predicts the dissociation process of the nitrogen molecule and hydrogen chain [39]. The neural network architecture could also be part of the calculation. It has been demonstrated in a recent study, in which the neurons act as the tight-binding (TB) matrix elements in the Hamiltonian parameterization of the TB model for energy band calculation [40]. From calculation methodology to case studies, several works report that machine learning-augmented DFT works efficiently with high accuracy for atomistic modeling of devices, including prediction of atomic force in phase change memory [41], calculation of potential energy surface in SiGe alloy [42], and simulation of surface reconstruction of the Si(111)−(7 × 7) surface [43].

**Fig. 7:**
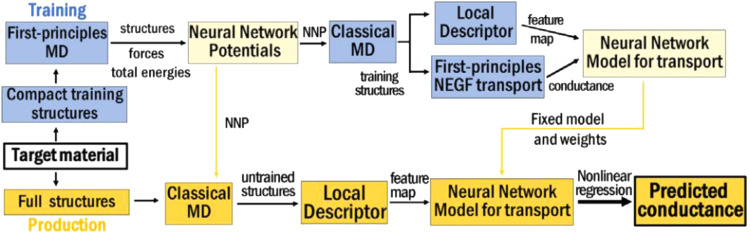
**The proposed NN**—**NEGF computation framework in reference**[44]**.**

In practical TCAD applications for the development of the 5 nm node and beyond, the quantum transport features and device merits are essential in simulations. Apart from the calculation of electrostatic properties, the quantum transport simulation using the non-equilibrium Green's function (NEGF) method is performed along with the machine learning method as reported in recent studies. M. Burkle et al. have proposed an NN—NEGF simulation framework (Fig. 7) that can predict the conductance of a large system qualitatively with considerable accuracy while the computational costs are only a fraction compared to those of conventional first-principle methods [44]. Furthermore, recently Y. Zhou et al. presented AD-NEGF, the first end-to-end differentiable NEGF model for quantum transport simulation where the numerical calculations are carried out in the deep learning framework Pytorch and the backward gradient is calculated efficiently using the proposed implicit layer technique [45]. The abovementioned examples show that the machine learning method is a strong candidate to resolve the bottleneck of the computation efficiency of first-principle methods for device analysis and optimization.

### 4.2 Semiclassical device simulation with ML

**Fig. 8:**
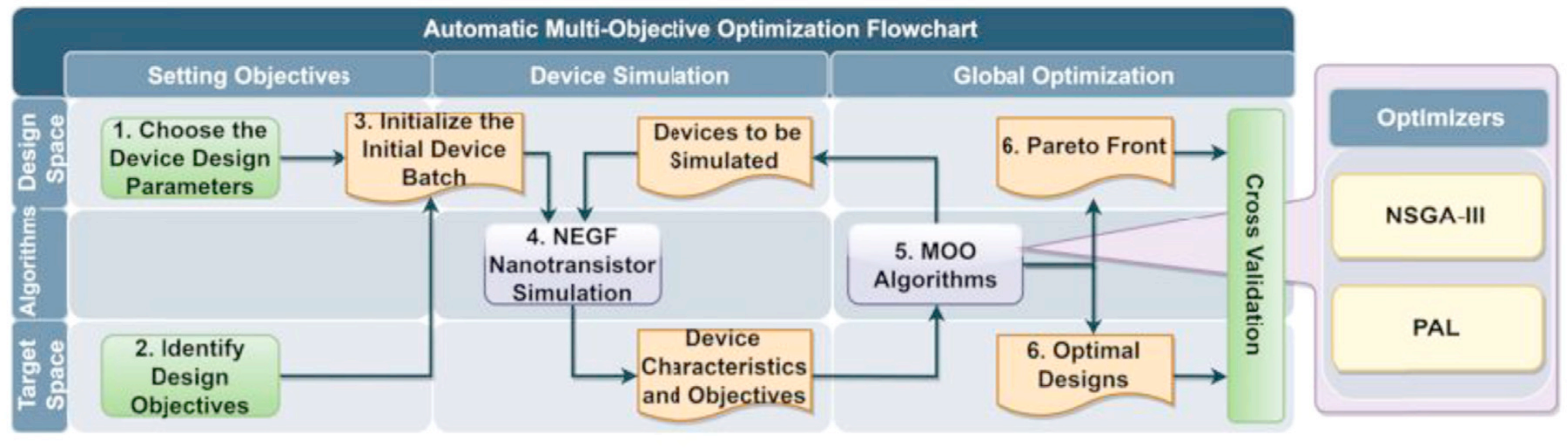
**The proposed MOO framework couple with machine learning for 2D TMDC and black phosphorene FETs**[55]**.**

Although a lot of progress in atomistic simulation with ML have been made in recent years, the computational costs are still too high for the massive simulation demand by industry. Bridging TCAD and ML in the DD framework, i.e., the semiclassical level, is more compatible to current sub-5 nm node development. To this end, the following updates have been proposed and addressed by several academic and industrial research groups. Traditional design of experiment (DOE) based on TCAD is time-consuming with a large number of calculations and a possible solution for this challenge is machine learning-based modeling. It has been frequently demonstrated in recent work that artificial neural network (ANN) has great capability in capturing nonlinear relationships with high accuracy between device parameters and electrical characteristics, promising a significant reduction of computation costs of predicting electrostatic potential [46,47], current-voltage (IV), and capacitance-voltage (CV) relationships [48], [49], [50] as well as threshold voltage [51], metal work function [52], and other figures of merit [53,54]. Furthermore, machine learning works well not only in device characteristics prediction but also in device optimization where machine learning is coupled with a multi-objective optimization algorithm that considers the trade-off between electrical characteristics carefully. For instance, a multi-objective optimization (MOO) framework was proposed with Pareto active learning to optimize 2-D transition metal dichalcogenide (TMDC) and black phosphorene FETs [55]. The 2D FET optimized by the proposed framework (Fig. 8) meets the International Roadmap of Devices and System target of 2025 and 2028 technology nodes.

## 5 Machine-learning engine compact model development

### 5.1 Development of ML assisted compact modeling

In 1992, the first MOS transistor model using a neural network was developed [56]. It featured an ANN and a unique continuous function covering all operation regions and showed that for such a black box modeling approach it is challenging to choose the mapping function and the optimization method for parameter extraction. Thereafter, this method was applied to various semiconductor FETs such as Microwave, RF-FETs, HEMT, advanced Si-MOSFET, TFT and multi-state devices.

***Microwave, RF-FET, and HEMT:*** In the late 1990s, due to challenges in microwave design [57], the standard multilayer perceptron neural network was applied to microwave transistor models characterizing DC currents [58], S-parameters [59], as well as noise parameters [60,61]. In 2003, an adjoint neural network method was proposed for sensitivity analysis in neural based microwave design, allowing training of neural models with not only input-output relationships but also their derivatives [62]. Thereafter, measurement-based neuro models (e.g., NeuroFET [63], DynaFET [64]) were developed by commercial companies and the MLCM methodology was proposed to accelerate parasitic-aware RF circuit analysis with a fast and accurate generation of compact distributed circuit models [65]. The ANN approach has been used for high electron mobility transistors (HEMT) [66] to characterize current characteristics, noise [67], and S-parameters depending on temperature and frequency [68]. In terms of self-heating, kink effects, and dynamic trapping [69], ANN-based electrothermal model were developed [70].

***Advanced Si-MOSFET and thin-film transistors:*** Emerging technology devices have reached sub-3 nm nodes and exhibit complicated physical phenomena as mentioned above. Several works of MLCM [71], [72], [73], [74], [75], [76] have been reported for advanced technology nodes to characterize device characteristics to achieve accurate DC, AC, or transient simulations. The working principles of thin film transistors differ from those of traditional MOSFETs, as evidenced by variations in surface potential and transport equations. Consequently, there is a need for increasingly specialized device model, which deviate significantly from industry standards. Considering the diversity of channel materials, ANN-based models for oxide-based transistors [77,78] and 2D-material-based transistors [79,80] have been developed. Noteworthy datasets using a generic NEGF method were obtained [80], showing potential in exploration of new materials.

***Multi-state devices:*** The diversity of multi-state devices, such as (anti-) ferroelectrics and memristors obstructs the development of a unified device model for different multi-state switching mechanisms based on the physics-driven method. Thus, a data-driven method such as MLCM is preferentially adopted to circumvent this issue. In contrast to modeling of conventional transistors, such devices depend on history and are characterized by state variables in specific compact models. Efforts to develop accurate memristive MLCM, such as generalized frameworks [81,82], process-aware models [83,84], and physics-ML hybridization [85] have been reported.

### 5.2 MLCM methodology

**Fig. 9:**
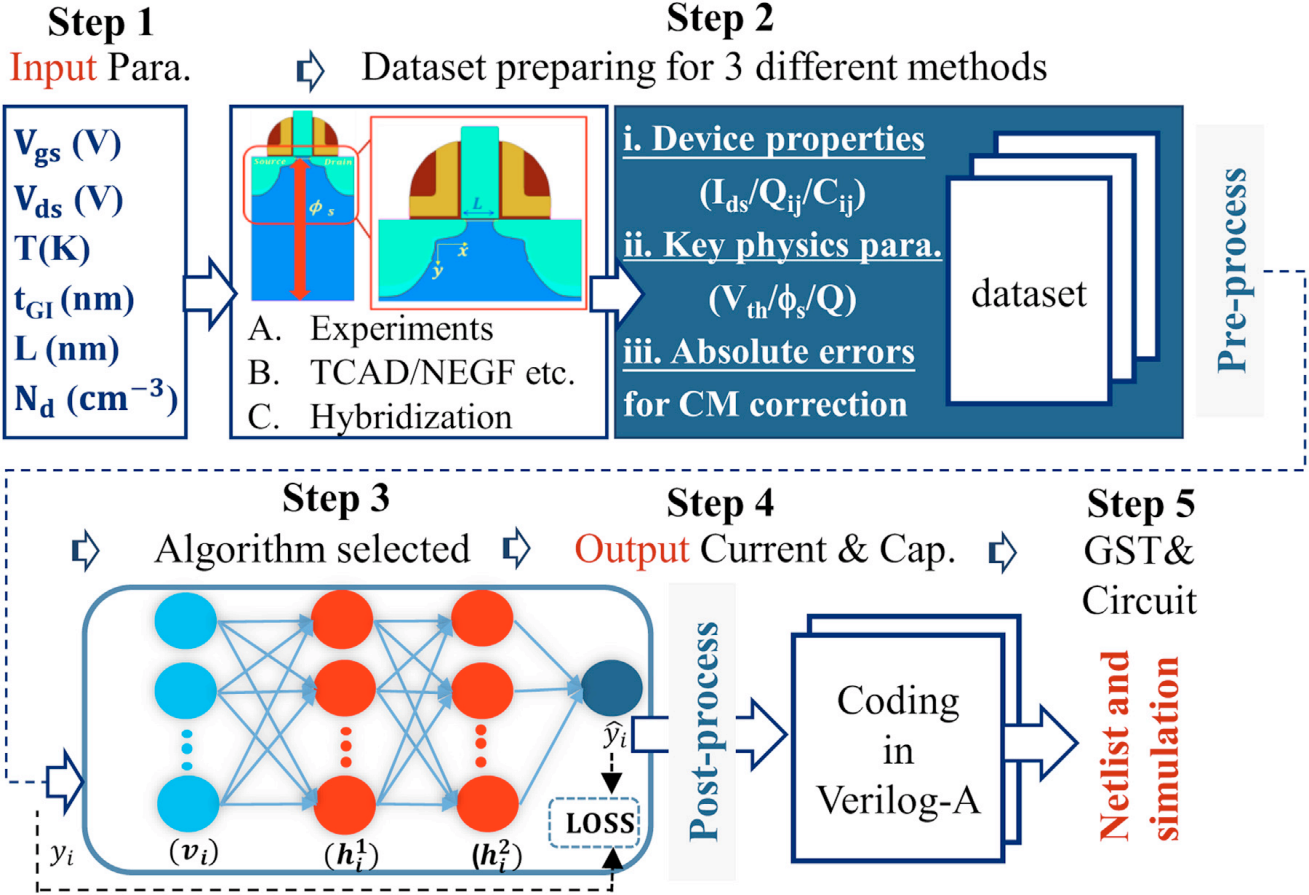
**The procedure of ML compact model development**.

A general framework for developing MLCM is presented with five generic steps, including (step 1) ***parameter*** determination, (step 2) ***dataset*** preparation, (step 3) ***algorithm*** selection, (step 4) ***Verilog-A*** coding, and (step 5) ***simulation*** of circuit, as shown in Fig. 9.

***Parameters*** are mostly determined by the device geometry, the applied voltage, and operation environment [71]. For transistors, basic parameters include voltage between terminals, temperature *T*, gate length *L*_gate_, gate-insulator thickness (*t*_GI_), and doping profiles (*N*_d_). Commonly, devices are measured at specific conditions, such as temperature and certain gate lengths. MLCM will possibly overfit if taking such parameters directly as inputs. To avoid this issue, a Taylor expansion assisted modeling can be adopted for expressing specific parameter dependence to reduce the ANN-model dimension [86].

***Datasets*** are inherently important in data-driven technology and can be obtained from experiment, simulation, or hybrid methods. For advanced devices such as sub-5 nm GAA-FETs, ultra-scaled vertical IGZO FETs, or Fe-SOI-FETs, development of MLCM based on experimental datasets is very limited. For such applications, device simulators (TCAD or NEGF) are employed to enlarge dataset-size under different parameter conditions. However, often there is a gap between experimental and simulated results, and the transfer of simulation-trained to experiment-trained models is a critical problem. Possible solutions for that are provided by algorithms such as transfer learning [87,88].

**Fig. 10:**
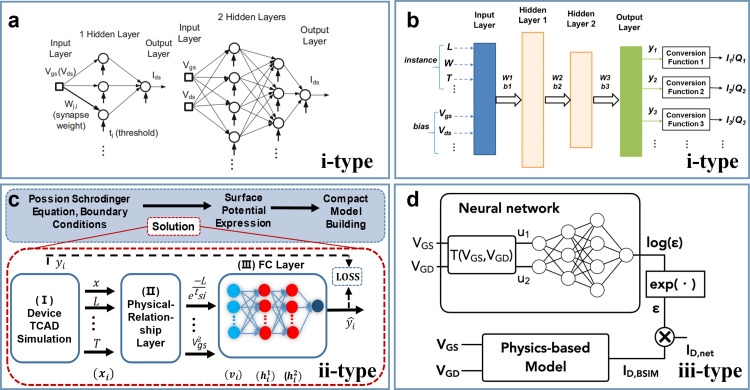
**Different types of datasets for training.** Feed-forward networks with voltage inputs and current output were used in (a) [73]. Conversion function was used to convert the output variables of the ANN in (b) [74]. TCAD simulation was used to obtain the data used to train the PRNN in (c) [71]. (d) shows the neural network design selecting the core BSIM-CMG as the starting physics-based model [76].

Different MLCM approaches (see Fig. 10), require different datasets for training which can be classified into 3 types as below:

***i-type:*** The data is of device properties consistent with outputs. Regardless of traditional CMs, experimental or simulated electrical characteristics are directly-used [73] or simply-converted [74].

***ii-type:*** The data is of key physics-based parameters of devices used for hybrid ML models. To obey the basic rules, a physics inconsistent issue should be taken into consideration. If referring to 3 major classical models, the key obstacle of CM is acquiring key parameters (i.e., *V*_th_/*ϕ*_s_/*Q*) of the channel in scaling-down advanced technology node through solving the Poisson's equation, even coupled with Schrödinger equation. Thus, the ii-type data driven models are on the foundation of traditional method by replacing the intractable part.

***iii-type:*** The data is of corrected term used for classical model correction. If keeping industrial standard models, iii-type data-driven models target at error-correction and accuracy-improvement, alternatively providing a method for quantum-corrected modeling.

***Preprocessing*** of datasets is quite important for efficiency and accuracy of compact models. Generally, *I*_ds_ spans several orders of magnitude (e.g., even *I*_on_/*I*_off_ ∼ 10^15^ for IGZO transistors), and numerical issues, e.g., the weight attribution [89], may occur during training processes. Similar to classical models, accuracy of both sub-threshold and above-threshold characteristics is challenging. To mitigate such issues and improving efficiency, strategies like initial MOSFET sizing [89], Latin hypercube sampling algorithms [90], and logarithm *I*_ds_/*V*_ds_ preprocessing [73] are proposed.

***Algorithm*** selection is critical in MLCM development. Multilayer perceptron (MLP) with backpropagation method has been widely used in semiconductor device compact modeling [71]. The available activation functions include ReLu, softsign, hyperbolic tangent, sigmoid, softplus, ISRU, ISRLU, and Swish. Specific hybrid activation functions of hyperbolic tangent and sigmoid are used to improve training accuracy [6,91]. Generally, Mean-Squared-Error (MSE) $\frac{\sum {\left(y_{i}-\hat{y_{i}}\right)}^{2}}{n}$ [92,93] is a loss function iteratively minimized with stochastic gradient descent [94]. In order to improve the accuracy of ANN models, a loss function with the error of derivatives of current or charge with respect to *V*_gs_ and *V*_ds_ was proposed [74]. To optimize the training process, a neuro-evolution-based efficient compact model was developed with a gradient-free algorithm [91]. Because of time-consuming parameter extraction processes, a Graph-based compact model was developed [95] which converts a conventional model into a set of nodes for key physical parameters [96]. Additionally, a multi-gradient neural network (MGNN) was used to demonstrate nonlinear properties and high order derivatives for 2D-material-based [97] and cold-source-based transistor [98]. Furthermore, table-based interpolation (TBI) [99], generalized moving least-squares (GMLS) [99], and feed forward deep neural networks (FFNN) [86] were employed for device modeling. For transient circuit simulation, the recurrent neural networks (RNNs) were adapted and transformed to a continuous-time model [100].

***Verilog-A*** is regarded as the *de facto* standard language for implementing compact models [101] in circuit simulator. Since Verilog-A language is not well-suited for matrix calculations, an automation script (Python [102] for example) can be used to perform this transformation process and realize the ANN algorithm, as shown in Table 1. Thus, matrix calculations are converted to basic multiplications compatible with Verilog-A.

**Table 1:** **The pseudo code for transforming ANN to Verilog-A language. Adapted from reference**[71]**.**

(Ⅰ) Parameter input for $w_{i}$
for$w_{i}$in framework with file.open($w_{i}$_value.txt) as f1:
for line in f1.readlines():
line_list = line.split() for j in range(len(line_list)):
*a*="parameter real$w_{i}$”+str(i)+’ =‘+str(line_list[*j*])+‘;’
f.write(a) *i*=*i*+1 *i*=1

**(Ⅱ)Forward propagation realization for**$h_{2}$

for i in range(1,len(hidden layer 2)):
*a*=‘$h_{2}$’+str(i)+‘=’+‘1/(1+exp(-(’
for j in range(1,len(hidden layer 1)):
*a*=*a*+‘$w_{2}$’+str(i)+str(j)+‘*$h_{1}$’+str(j)+‘+’
*a*=*a*+‘$b_{2}$’+str(i)+‘;’ *a*=*a*+‘)’ f.write(a)

***Simulations*** are conducted with Verilog-A coding. However, circuit simulations may fail to converge for some MLCM methodologies, due to unintended negative values or high-order derivatives of terminal currents or charges. Therefore, in analogy to traditional compact modeling methods, Gummel symmetry tests (GST) are required to benchmark the continuity and convergence. To this date, merely a few publications [74,103] have reported this standard test.

### 5.3 Physics-aware technology

As mentioned above, a key problem of data-driven modeling methodology are physical inconsistencies. Obeying the basic physical rules (for example ohm's rule), the drain-source current should equal to zero at *V*_ds_ = 0. Moreover, beyond the voltage range used for training, the predicted results should converge continuously. To meet above requirements, NN models can be made physics-aware through algorithm optimization or prior-knowledge.

**Fig. 11:**
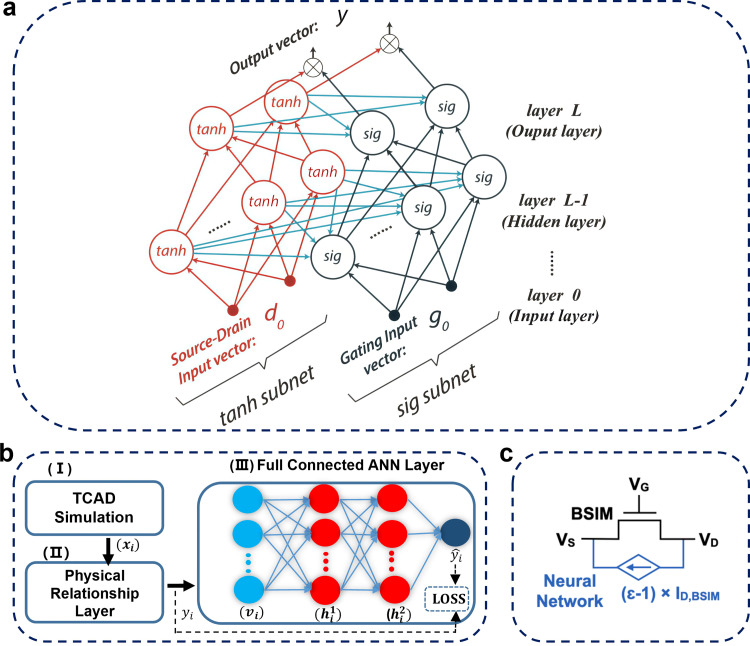
**Physics-aware technology.** (a) is adapted from reference [6], (b) is adapted from reference [71], and (c) is adapted from reference [76].

***Algorithm optimization*** enhances the physics-awareness of NN models. One approach to achieve this is to treat CM as a regression problem, leading to PINN algorithm [6]. From physics-based view device working principles vary under different applied voltages. Accordingly, NN architecture can be split into a tanh subnet with source-drain input vector and a sig subnet with gating input vector, as shown in Fig. 11a. By improving the algorithm, output characteristics become more consistent with the underlying physics.

***Prior knowledge*** of basic physics rules to assist NN models is another method to solve the issue.

For i-type NN models, a simple conversion based on Ohm's law would read

(5) $$\begin{array}{c} I_{\text{ds}}\;=\;I_{0}\cdot V_{\mathrm{ds}}\cdot 10^{y} \end{array}$$

where *y* is the output from ANN models. In this expression, the current will be forced to be zero at *V*_ds_ = 0. Other physics-aware methods have been introduced in the literature [95,104].

For ii-type NN models adapted from conventional CM development process, a physics-relationship-neural-network is proposed which includes a physical-relation layer to account for basic physical prior knowledge to better capture data nonlinearity. The physical layer definition reads

(6) $$\begin{array}{ccc} v_{i} & = & [V_{\text{gs}},V_{\text{ds}},N_{d},T,x,e^{\lambda \frac{x-L}{t_{\text{Si}}}},e^{\lambda \frac{-x}{t_{\text{Si}}}},V_{\mathrm{ds}}e^{\lambda \frac{-L}{t_{\text{Si}}}},V_{\text{bi}}\left(1-e^{\lambda \frac{-L}{t_{\text{Si}}}}\right),\\ & & \;V_{\text{gs}}^{2},V_{\text{gs}}-V_{\text{ds}},\text{log}\left(\frac{L_{\text{Di}}V_{\text{gs}}}{t_{\text{Si}}}\right)] \end{array}$$

where $v_{i}\in R^{12\times 1}$, $\lambda \;=\;\sqrt{\frac{\varepsilon_{\text{ox}}t_{\text{Si}}}{T_{\text{ox}}\varepsilon_{\text{Si}}}}$, $L_{\text{Di}}\;=\;\sqrt{\frac{\varepsilon_{\mathrm{Si}}v_{t}}{qn_{i}}}$is the intrinsic Debye length, and$V_{\text{bi}}\;=\;V_{t}\cdot \ln\left(\frac{N_{\text{sd}}N_{d}}{n_{i}^{2}}\right)$ is the build-in potential at source/drain terminal. Min-Max normalization is then applied to standardize the data $v_{i}$. With fundamental relations of physics [105], the sample complexity can be further reduced. Using trained surface potential, a hybrid model can be expressed as follows:

(7) $$\begin{array}{c} I_{\text{ds}}\;=\;\mu_{\text{eff}}C_{\text{ox}}\frac{W}{L}\left[V_{\text{gf}}\cdot \left(\phi_{\text{sd}}-\phi_{\text{ss}}\right)-\frac{1}{2}\left(\phi_{\text{sd}}^{2}-\phi_{\text{ss}}^{2}\right)\right] \end{array}$$

The effective channel mobility$\;\mu_{\text{eff}}$ is given by

(8) $$\begin{array}{c} \;\mu_{\text{eff}}\;=\;\frac{\;\mu_{0}}{1+\text{MUE}\cdot {E_{\text{eff}}}^{\text{THEMU}}+\text{CS}\cdot {\left(\frac{q_{d}}{q_{d}+q_{i}}\right)}^{2}} \end{array}$$

where $\;\mu_{0}$ is the low-field mobility, the parameters MUE and THEMU account for the mobility degradation by the effective vertical field (i.e., $E_{\text{eff}}$), and CS introduces Coulomb scattering with $q_{b}$ and $q_{i}$, which represent the normalized charge of the depletion region and inversion charge [106]. And $V_{\text{gf}}$ is given by

(9) $$\begin{array}{c} V_{\text{gf}}\;=\;\text{ln}\left[1+e^{\left(V_{\text{gs}}-V_{\text{fb}}+2V_{t}\right)\cdot \text{Sl}}\right]/\text{Sl} \end{array}$$

where Sl acts as the correction parameter to the subthreshold swing.

The surface potential obtained from ANN at source side is given by

(10) $$\begin{array}{c} \phi_{\text{ss}}\;=\;\phi_{s}\left[V_{\text{gs}},V_{\text{ds}},T,\cdots ,x\right]-\phi_{s}\left[0,0,T,\cdots ,x\right] \end{array}$$

(11) $$\begin{array}{c} \phi_{\text{sd}}\;=\;\phi_{\text{ss}}+V_{\text{dsx}} \end{array}$$

(12) $$\begin{array}{c} V_{\text{dsx}}\;=\;gV_{\text{ds}}\cdot {\left(1+{\frac{gV_{\text{ds}}}{V_{\text{dsat}}}}^{m}\right)}^{-\frac{1}{m}} \end{array}$$

where $\phi_{\text{sd}}\;=\;\phi_{\text{ss}}$ and the current equals to zero at *V*_dsx_ = 0 in (7), (8), (9), (10), (11), (12). Moreover, this method is more compliable to other characteristic expression (e.g., terminal charge/capacitance) given by

(13) $$\begin{array}{c} Q_{g}\;=\;W\times C_{\text{ox}}\int_{0}^{L}\left(V_{g}-V_{\text{fb}}-\phi_{s}\left[V_{\text{gs}},V_{\text{ds}},T,\cdots ,x\right]\right)dx\;\;\; \end{array}$$

(14) $$\begin{array}{c} Q_{d}\;=\;W\times C_{\text{ox}}\int_{0}^{L}q_{i}\cdot \frac{y}{L}dx \end{array}$$

(15) $$\begin{array}{c} Q_{s}\;=\;W\times \int_{0}^{L}q_{i}\left(1-\frac{y}{L}\right)dx \end{array}$$

(16) $$\begin{array}{c} C_{\mathrm{mn}}\;=\;-\frac{\partial Q_{m}}{\partial V_{n}},\;m\;\neq \;n;\;C_{\mathrm{mm}}\;=\;\frac{\partial Q_{m}}{\partial V_{m}} \end{array}$$

For iii-type NN models, considering the zero-problem and symmetric devices, the correction function must satisfy the following equation:

(17) $$\begin{array}{c} I_{\mathrm{ds},\mathrm{BSIM}}\left(V_{\mathrm{gs}},V_{\mathrm{gd}}\right)\times \varepsilon \left(V_{\mathrm{gs}},V_{\mathrm{gd}}\right)\;=\;-I_{\mathrm{ds},\mathrm{BSIM}}\left(V_{\mathrm{gd}},V_{\mathrm{gs}}\right)\times \varepsilon \left(V_{\mathrm{gd}},V_{\mathrm{gs}}\right) \end{array}$$

where the solved correction factor will obey the basic rules, and a connection with BSIM model is shown in Fig. 11c.

### 5.4 ML assisted process-aware and failure analysis technology

In analogy to traditional statistical compact models, process-aware simulations are important for ultra-scaled devices subject to manufacturing variability. With massive processing data in the advanced technology nodes, the statistical effects are necessarily introduced in MLCM. Particularly for memory devices, the distribution of high or low resistance states are important for circuit performance analysis. Such methods need to obtain standard deviations of process parameters, and the mean/variance propagate through ANN. Various aspects of statistical compact model using ANN have been reported in literature [83,84,[107], [108], [109]]. On the other hand, in the case of few-shot learning required for advanced fabrication devices (e.g., GAA-FET), transfer learning was proposed as a solution to failure analysis for 3 nm beyond CMOS transistors [49].

### 5.5 ML assisted parameter extraction

As mentioned above, the parameter extraction method is one of the bottlenecks of advanced device compact models with a large number of parameters. A ML assisted parameter extraction method was proposed to tackle this problem for the BSIM-CMG model [110], which is a promising technique for efficient calibration.

## 6 Challenges of machine-learning engine TCAD-to-SPICE

The data-driven compact modeling is a *black-box* method, benefitting from massive data collections coming from advanced fabrication and semiconductor simulation. This approach unifies emerging devices even with different work principles. However, compared to general physics-driven compact modeling methods, it still faces some critical challenges.

***Unphysical problems:*** Due to a large *I*_on_/*I*_off_, accurate prediction is difficult in both sub- and above-threshold regions. Particularly, due to basic laws, the channel current should be zero at *V*_ds_ = 0. Beyond trained voltage regions, the curves should also meet physics-based requirements. By updating algorithms or employing prior-knowledge, various MLCM flows have been proposed to tackle such unphysical problems.

***Sub-model development:*** Beyond modeling current characteristics, developing sub-models for charge, capacitance, and other extrinsic effects remains challenging. At the time of writing, this is addressed by introducing an additional network and train selected datasets for i-type models, while ii-type and iii-type models adapted from traditional models mitigate this issue. However, the root problem are physical inconsistencies as real parameter relations are not included directly in common MLCM.

***Lacking industrial standard:*** GST has been a benchmark industry standard for MOSFET models and is barely used in the ML assisted models. Thus, a common industrial standard is highly required for MLCM to confirm their convergence and continuity for circuit simulation.

***Algorithm realization:*** Although basic matrix manipulations can be conducted by Verilog-A, it lacks an AI-orientation package for algorithm realization. In general, to realize MLCM through a complicated algorithm will require more resources than the common compact modeling method.

## 7 Conclusion

Emerging device model development has been systematically reviewed from device-physics-engine to machine-learning-engine. As scaling of advanced devices continues, the traditional physics-driven models (i.e., threshold voltage based, surface potential based, and charge based models) face challenges such as near-threshold modeling, increased complexity, atomic physics, reliability characterization, Hetero-integration IC, and extreme environment which obstruct accurate model development and efficient parameter extraction flows. To address that, machine learning methods were proposed to characterize semiconductor devices regardless of the complexity of the device physics. Such models harness the universal approximation power of artificial neural networks, capable of learning from massive simulation and experimental data. It automates generation of generic MOSFET compact models through a procedure of (step 1) parameter determination, (step 2) dataset preparation, (step 3) algorithm selection, (step 4) Verilog-A coding, and (step 5) simulation of circuit. This promising solution for semiconductor modeling can also boost TCAD-to-SPICE flows which are of increasing importance for DTCO. However, compared to industry-standard models, several challenges have emerged related to physical consistency, sub-model development, the absence of industry-wide standards, and algorithm implementation. Remarkable developments to tackle these issues were categorized into i-type, ii-type, and iii-type models, which differ in selected datasets and current expressions, by updating algorithm or employing prior-knowledge. With a hybrid method combining physics and data, an accurate, unified, and automated MLCM development not only shortens the gap between theory and manufacturing, but is also expected to be a strong candidate to accelerate the flow of design, simulation, and optimization of very large-scale circuit system.

## Declaration of competing interest

The authors declare that they have no conflicts of interest in this work.
